# Computational Aerodynamic Analysis of a Micro-CT Based Bio-Realistic Fruit Fly Wing

**DOI:** 10.1371/journal.pone.0124824

**Published:** 2015-05-08

**Authors:** Joshua Brandt, Graham Doig, Naomi Tsafnat

**Affiliations:** 1 School of Mechanical and Manufacturing Engineering, UNSW Australia, Sydney, New South Wales, Australia; 2 Aerospace Engineering Department, California Polytechnic State University, San Luis Obispo, California, United States of America; University of Zurich, SWITZERLAND

## Abstract

The aerodynamic features of a bio-realistic 3D fruit fly wing in steady state (snapshot) flight conditions were analyzed numerically. The wing geometry was created from high resolution micro-computed tomography (micro-CT) of the fruit fly *Drosophila virilis*. Computational fluid dynamics (CFD) analyses of the wing were conducted at ultra-low Reynolds numbers ranging from 71 to 200, and at angles of attack ranging from -10° to +30°. It was found that in the 3D bio-realistc model, the corrugations of the wing created localized circulation regions in the flow field, most notably at higher angles of attack near the wing tip. Analyses of a simplified flat wing geometry showed higher lift to drag performance values for any given angle of attack at these Reynolds numbers, though very similar performance is noted at -10°. Results have indicated that the simplified flat wing can successfully be used to approximate high-level properties such as aerodynamic coefficients and overall performance trends as well as large flow-field structures. However, local pressure peaks and near-wing flow features induced by the corrugations are unable to be replicated by the simple wing. We therefore recommend that accurate 3D bio-realistic geometries be used when modelling insect wings where such information is useful.

## Introduction

Past research has shown that most insects are only capable of flight through several unsteady effects induced by flapping their wings [1–6]. It is generally agreed that the most important effect is the development of the leading edge vortex, and the resulting delayed stall [2, 3, 5–8]. Due to rising interest in micro unmanned flight vehicles (MUAV), a quantified understanding of insect flight is of great importance [9–11].

Some of the major challenges in the analysis of insect flight are the design of appropriate modeling and recording techniques, and the difficulty in acquiring accurate experimental force measurements [2, 7]. Quantification of insect flight aerodynamics is difficult due to the relative size scale of insects to other more conventional flying bodies, coupled with the high frequencies associated with wing flapping.

Experimental and numerical approaches for modeling and recording of insect flight have been developed utilizing 3D infrared high-speed video live recording [4, 12], wind tunnel testing [13–16], particle image velocimetry (PIV) [17] and experiments based on dynamically scaled models [4, 8, 18, 19]. Computational fluid dynamics (CFD), a common technique for numerical aerodynamic analyses, has been used to study insect flight in 2D and 3D, simulating both flapping and gliding flight over a range of Reynolds numbers [3–6, 20–22]. As summarized in Table 1, such CFD simulations often use simplified geometry to represent the wing to reduce computational complexity.

**Table 1:** Summary of numerical simulations reported in the literature.

Author	Re Range	Wing Type	Cross Section	2D/3D	Flight style
Aono et al. (2008) [3]	134	Fruit Fly wing	Elliptical	3D	Flapping
Sun and Tang (2002) [5]	136	Fruit Fly wing	Elliptical	3D	Flapping
Wu and Sun (2004) [6]	20–1800	Fruit Fly wing	Constant thickness (flat)	3D	Flapping
Du and Sun (2012) [20]	800	N/A	‘Saw-tooth’ approximation	3D	Flapping
Meng et al. (2011) [21]	35–3400	N/A	‘Saw-tooth’ approximation	2D	Flapping
Vargas et al. (2008) [22]	500–10000	Dragonfly wing	Bio-realistic	2D	Gliding
Meng and Sun (2013) [23]	200–2400	N/A	‘Saw-tooth’ approximation	2D	Gliding

More recent studies have also made use of X-ray micro computed tomography (micro-CT) for both structural insect wing investigation [24] and the development of an at-scale physical hoverfly wing model [25]. Micro-CT is a non-destructive imaging method which allows visualization of the three-dimensional structure of specimens. Micro-CT has been used extensively in the study of biological materials [26], and is often combined with numerical modeling techniques such as finite element analysis [27]. The digitized micro-CT visualization dataset can be used to create high fidelity 3D geometry for CFD analyses. In this way the exact 3D structure of the specimen is modeled and no simplifying assumptions are made in regards to its geometry.

Tanaka et al. (2011) [25] outline various issues in the manufacture of at-scale wing models in discussing their experiments on hoverflies. Torsional flexibility and flexural compliance of the wing surface are important features; the structure, inertial response, frequency modes, and trajectories of an at-scale model are similar to those of insects in free flight, therefore precise reproduction is highly advantageous. The CT-derived hoverfly wing—larger and more structurally complex than a fruit fly wing—used to make at-scale models in that study featured elliptical cross-sectional veins incorporated into the surface of a 3D CAD model based on micro-CT.

While evidence suggests that the corrugated nature of wings is extremely beneficial for preserving structural integrity during flight [24, 28, 29], in most cases the corrugations are observed to have detrimental effects on aerodynamic performance when compared to a flat plate [20–22, 29].

Interestingly, Vargas et al. (2008) [22] found that at low angles of attack, the corrugations of the wing induce pockets of recirculation, and thus sections of negative shear drag. At Reynolds numbers greater than 5000, the corrugated dragonfly wing section was shown to have better performance than a flat plate, despite its unconventional profile. Meng and Sun’s (2013) [23] later CFD simulations corroborated the finding of induced negative shear drag; however the corrugations consistently reduced aerodynamic performance. This was consistent with Vargas et al.’s (2008) work given that the highest tested Reynolds number was 2400.

Despite the extensive studies already produced in the computational field, very few have addressed the fact that the cross-section of an insect wing changes significantly along the span. As such, it is possible this approximation is a source of numerical error in these analyses. Like other insects, *Drosophila virilis* wings exhibit a complex 3D corrugated wing structure.

The *Drosophila* has a very well-defined series of veins and vein-junctions, with relatively little spatial variation—standard deviation of junction points from the mean is a few percent of the wing reference chord or span—in their position even over thousands of samples [30], one can expect the wing produced here to be a highly representative shape. While this would also make the wing relatively easy to approximate from recent data, the micro-CT approach is able to produce every nuance—that is, the statistical anomalies of the wing are preserved far more accurately. At the same time, micro-CT derived methods show promise as a technique to eventually determine the material properties and therefore the aeroelastic behavior of the wing in realistic flapping conditions. The present study represents an initial step of reconstructing the wing for initial aerodynamic analysis, for preliminary insight into the 3D aerodynamic characteristics of the wing at ultra-low Reynolds numbers which have been relatively unstudied due to difficulties in producing meaningful experimental results. To the best of the authors’ knowledge, this is the first micro-CT based CFD aerodynamic analysis of an insect wing.

## Materials and Methods

### Micro-CT

A specimen of the fruit fly *Drosophila virilis* was acquired and stored frozen for approximately 2 weeks prior to scanning. The specimen was imaged in a SkyScan 1172 high resolution desktop microCT (Bruker microCT, Kontich, Belgium) at 40 keV and 250 μA, with a voxel resolution of 4.9 μm^3^. The specimen was rotated around 180° at angular increments of 0.20°. The microCT radiograph projections were reconstructed (NRecon version 1.4.4) to obtain an axial slice image dataset. Three-dimensional rendering was performed using CTVox (version 2.3.0.0, Bruker microCT, Kontich, Belgium). The 8-bit (256 greyvalues) image dataset was imported into AMIRA (version 5.4.2, Visualization Sciences Group, Burlington, USA) for image extraction and segmentation.

**Fig 1:**
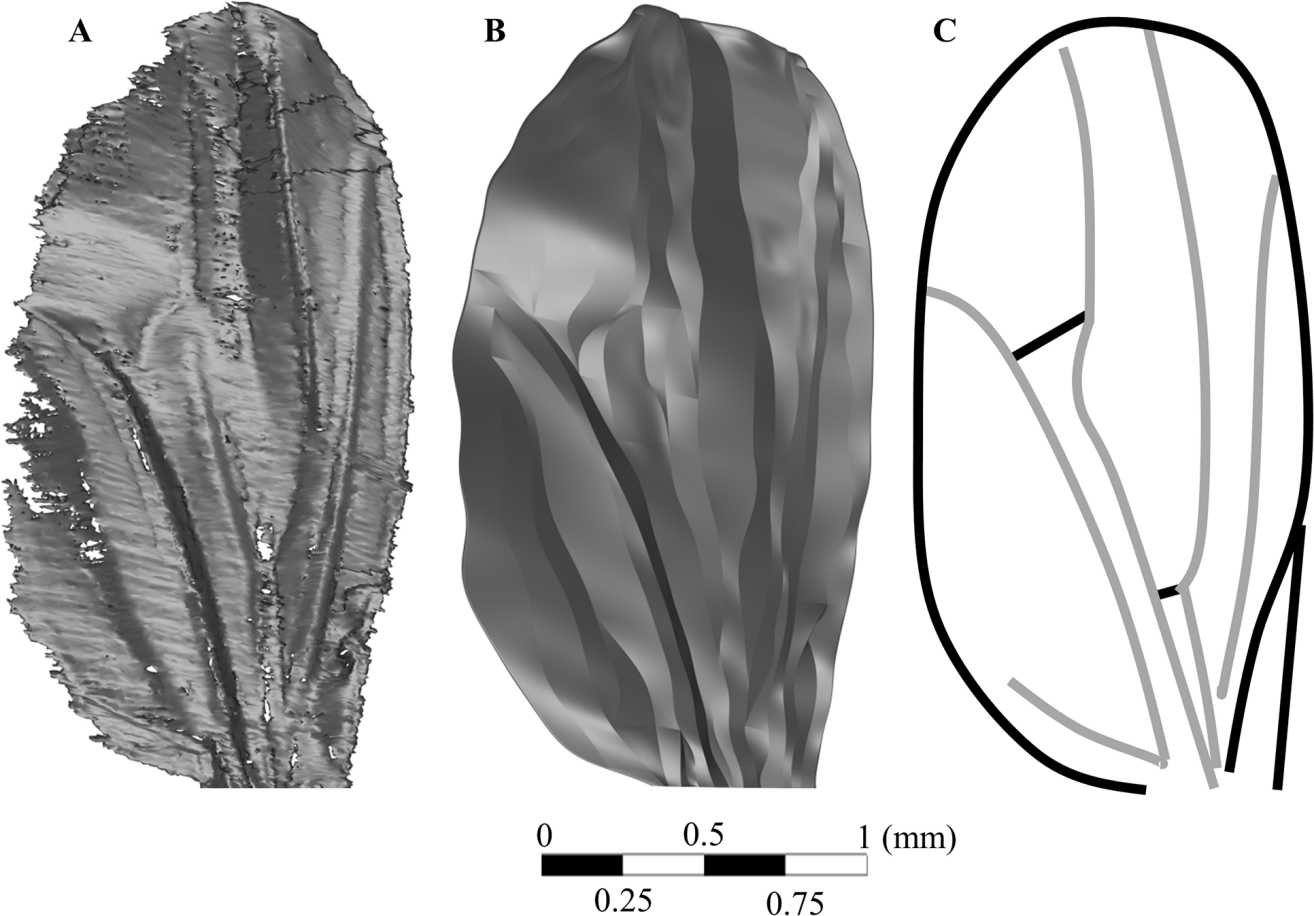
Reconstructed micro-CT wing and inherent vein structure. A) The initial surface obtained from post-processing of the Micro CT scan, B) the “cleaned” surface of the reconstructed wing in CAD required for the CFD software, and C) an approximation of the vein structure (based on Houle et al, 2003 [30]) indicating primary regions of structural strength which also define the corrugations. Interpolation of missing data was required in order to achieve a smooth surface usable in CFD.

The tomographic dataset was imported into CATIA (version 5 revision 20, Dassualt Systems, Velizy-Villacoublay, France) and resurfaced to replace missing data for use in CFD, as shown in Fig 1. It was found that the wing was subject to geometric twist averaging at 10° along the span. This data was also utilized to create a simple flat wing model which preserved the leading and trailing edges, along with the geometrical twist, but did not contain any corrugations.

### CFD

A commercially-available and widely-used Reynolds-Averaged Navier Stokes three dimensional finite-volume solver, ANSYS CFX, was used to produce all the numerical results presented here. Given an average chord length of 1.127mm, and inlet velocities of 1.051m/s, 2.103m/s and 2.943m/s, the respective Reynolds numbers tested were 71, 143 and 200. Reynolds number was calculated by:

$$\text{Re}=\frac{\bar{c}\times u_{{}_{\infty }}}{\nu }$$

Where $\bar{c}$ is average chord length, u_∞_ is the freestream velocity and *v* is the kinematic viscosity of air taken at 25°C.

As the simulation was of gliding and not flapping, the relatively low Reynolds numbers tested allowed a laminar, incompressible treatment of the full flowfield. All simulations were completed as steady-state. To ensure satisfactory accuracy at reasonable computational expense, second order discretization was implemented with node-based evaluation of flow variable gradients. An implicit, segregated, pressure-based solver was used to march towards steady-state solutions, defined as being attained only when the delta of lift and drag forces acting on the wing was negligible with extensive continued iteration. It was found that setting the RMS residuals to a convergence level of 1e^-8^ satisfied the convergence criteria. No quasi-unsteady vortex shedding was observed in the simulations. Mesh convergence was tested on the bio-realistic wing, and an identical meshing strategy was then applied to the simplified wing.

The total fluid domain consisted of a rectangular bounding region of dimensions 15c width, 12c height, and 8c depth—differences in reported forces of <1.1% were obtained with a considerably larger domain of 20c in all directions, thus the chosen domain was deemed suitable.

A hybrid meshing strategy was selected due to the ability to efficiently produce high-resolution surface meshes with prismatic growth layers to define the boundary layer in the near-wall region. This semi-structured region blended to a far-field unstructured tetrahedral mesh which allowed the overall cell count to be kept reasonable by growing cell sizes away from regions of strong flow gradients. A verification procedure was followed in which the mesh resolution was increased (non-linearly, focusing on the near-wing region) until the forces on the wing ceased to change beyond a set criteria of 0.25% with increased mesh cell density, indicating mesh-independence of the results. The final meshes used to produce all results discussed in the following sections consisted of 5.03 million cells, with the maximum size of mesh tested consisting of 7.74 million cells.

## Results and Discussion

### Validation

The numerical approach and wing characteristics were part-validated through a comparison to experimental results from the literature [14] in which the wings from a *Drosophila virilis* fruit fly were removed and attached to a 4 mm diameter wire. During the attachment process, it was noticed that some wings remained ‘flat’, while others retained a cambered profile similar to wing deflection during down-stroke. This gave rise to two different sets of experimental results for the flat and cambered profiles respectively.

**Fig 2:**
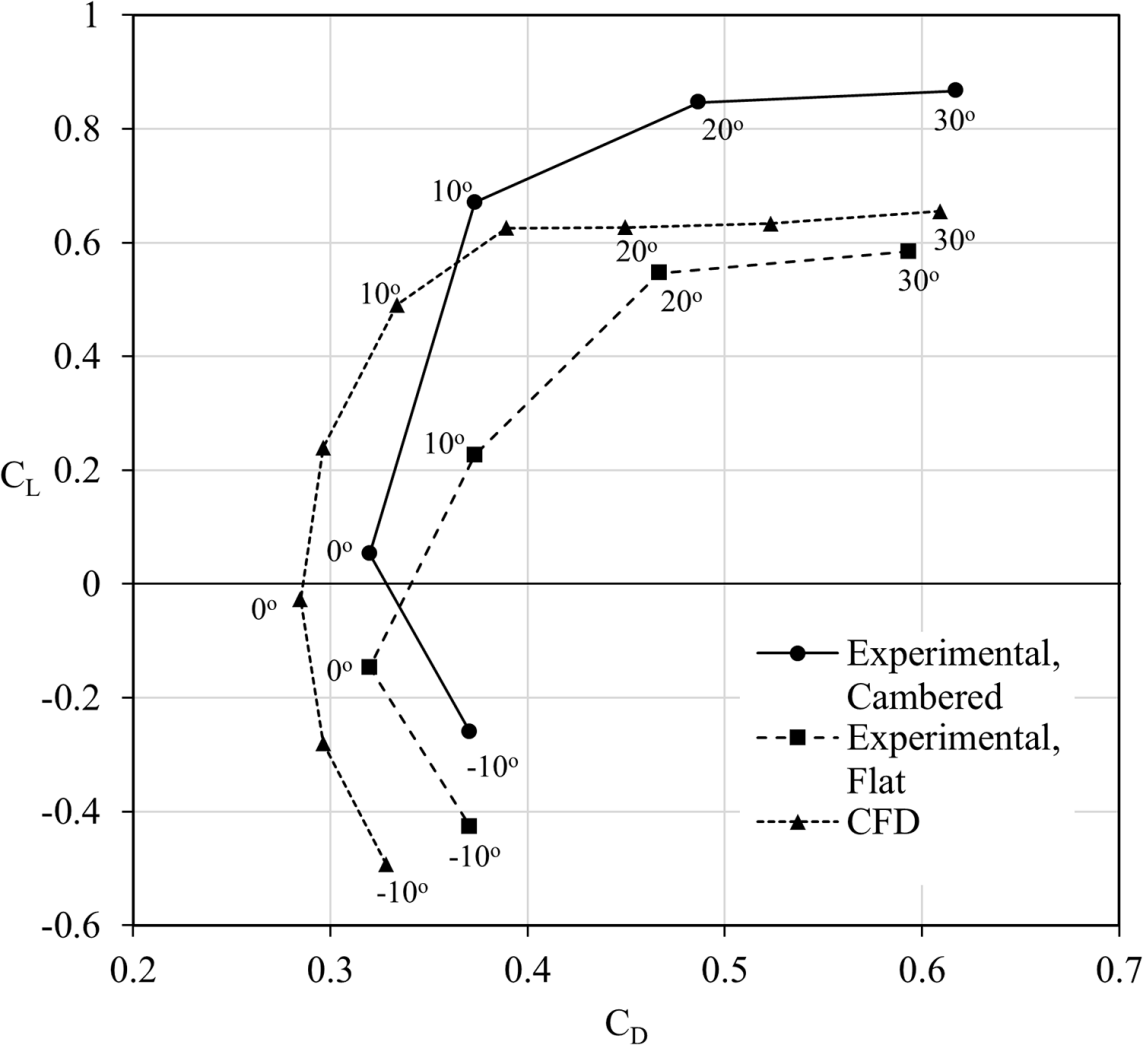
Aerodynamic performance comparison of experimental and computational results. A lift-to-drag (L/D) comparison between experimental results as taken from literature [14] and numerical results determined by computational fluid dynamics (CFD). Two sets of experimental results exist due to differing profiles (cambered and flat) being observed in testing [14]. The CFD results agree with the general trend of the experimental results, and are within expected values at higher angles of attack. Computational results made use of the bio-realistic wing for fair comparison to experimental results.

The CFD results follow the same trends, along with being within the expected range of values at higher angles of attack. Discrepancies in drag apparent at lower angles of attack may be in part explained due to the differences between the experimental setup and CFD model, yet CFD simulation data closely matches the experimental result trend. Fig 2 presents performance data defined by the lift to drag ratio.

Though every effort was made to ensure accuracy, the bio-realistic wing model used for the CFD analysis has a unique geometry, constructed from micro-CT scans, and is hence different from the wings used in the experiments. This includes the fact that the degree of camber in the wings used for experimentation is somewhat vaguely defined, and could not be accounted for appropriately. The CFD analysis also assumes the wing is a completely smooth no-slip wall, and does not apply any surface roughness to account for any possible micro-hairs. Finally, fruit fly wings will deform under aerodynamic loads, which was not included in the CFD model. At high angles of attack, the large degree of separation is the major defining influence, and hence the numerical values are of much higher correlation to the experimental results.

**Fig 3:**
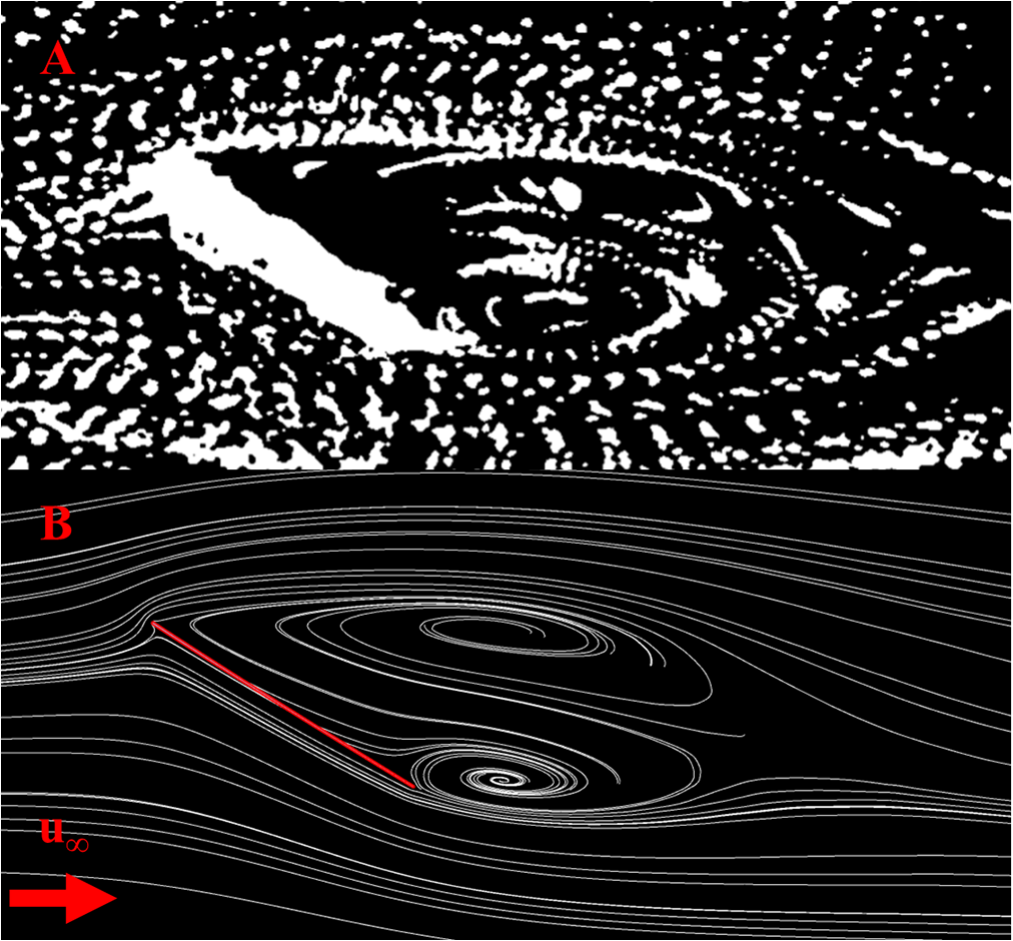
Flow-field comparison between experimental and computational results. Flow field comparison at a Reynolds number of 120 and an angle of attack of 31°, showing A) experimental results presented by Vogel (1967) [14] and B) numerical results for the flat wing as simulated by CFD. Both sets of results show a dual vortex system, with good correlation between the experiment and CFD simulation. The CFD flow field was taken at half span.

Fig 3 compares flow features observed at an angle of attack of 31° between Vogel’s (1967) constructed flat plate, and the CFD simulated simple wing. The simple wing flow features were taken from half span of the wing. The CFD results correlated well with the experimental results. The dual vortex structure is readily apparent in both images, and both images present similar sized vortices.

### Aerodynamic Analysis

#### Aerodynamic coefficients

**Fig 4:**
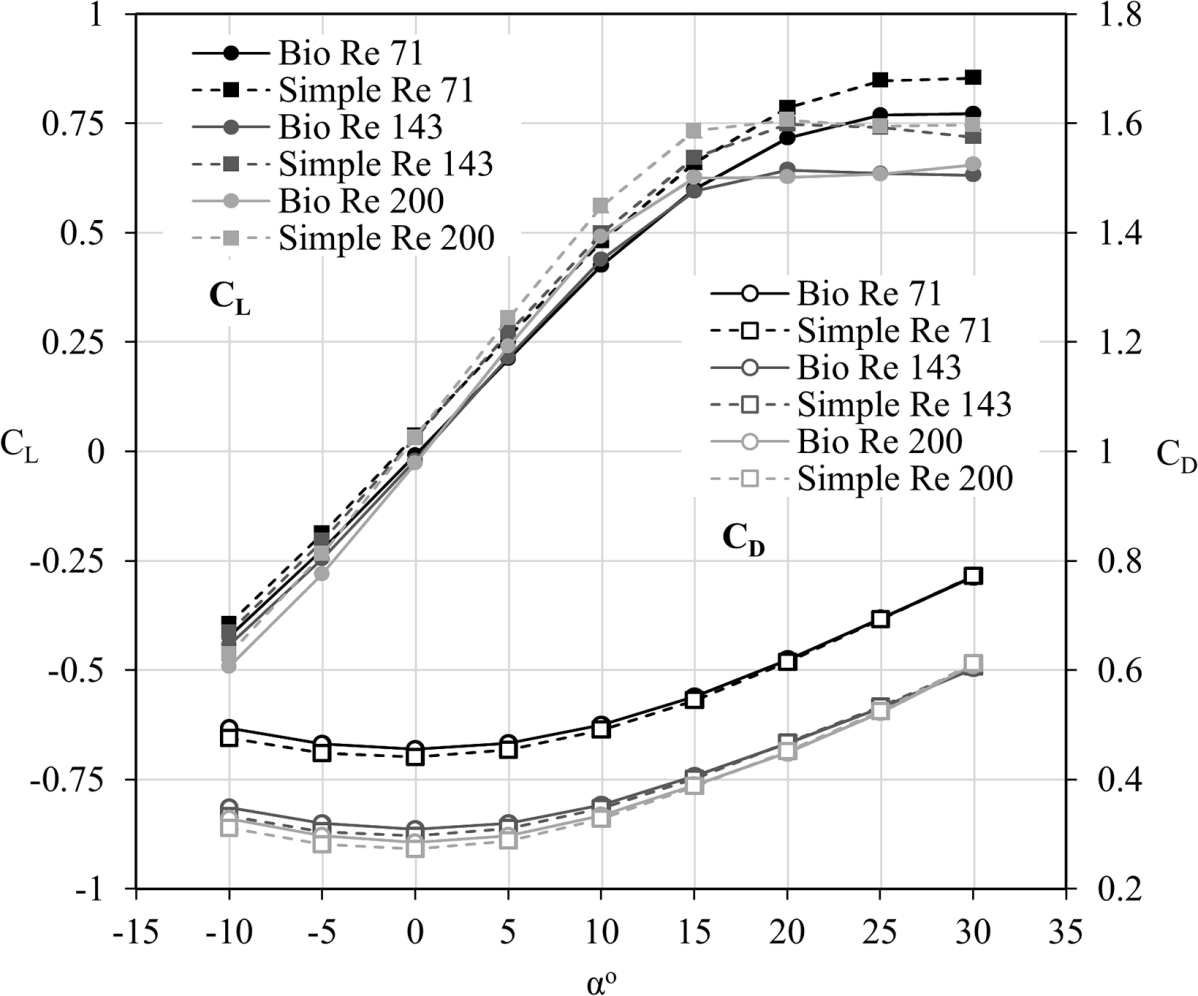
Lift and drag coefficients against angle of attack for the bio-realistic and simple wings. The lift coefficients (C_L_) and drag coefficients (C_D_) for the bio-realistic (bio) and simple wings against angle of attack (α°). Both wings share very similar performance trends. The lift is noted to plateau at about 15°. The flat wing presents a higher lift value regardless of Reynolds number. At large angles of attack the drag values match very well between the two wings due to the large degree of separation which minimizes the effects of the corrugations.

Comparisons between the flat and corrugated wing are shown in Fig 4 for lift and drag coefficients vs. angle of attack up to 30°. The Reynolds number was varied from 71 to 200, and clear evidence of enhanced lift and reduced drag is evidenced with incremental increases to Re. The differences in drag exhibit systematic trends across the range of angles, with a relatively similar enhancement to performance with higher Reynolds numbers. The lift curve indicates that the wing, although technically stalled at the highest angles since the flow is completely detached (observed in later sections), has not experienced lift-loss due to the significant low pressure associated with the large vertical structure emanating from the leading edge. This was not unexpected as previous works suggest this plateau can exist to up to angle of attack of 50° [14].

**Fig 5:**
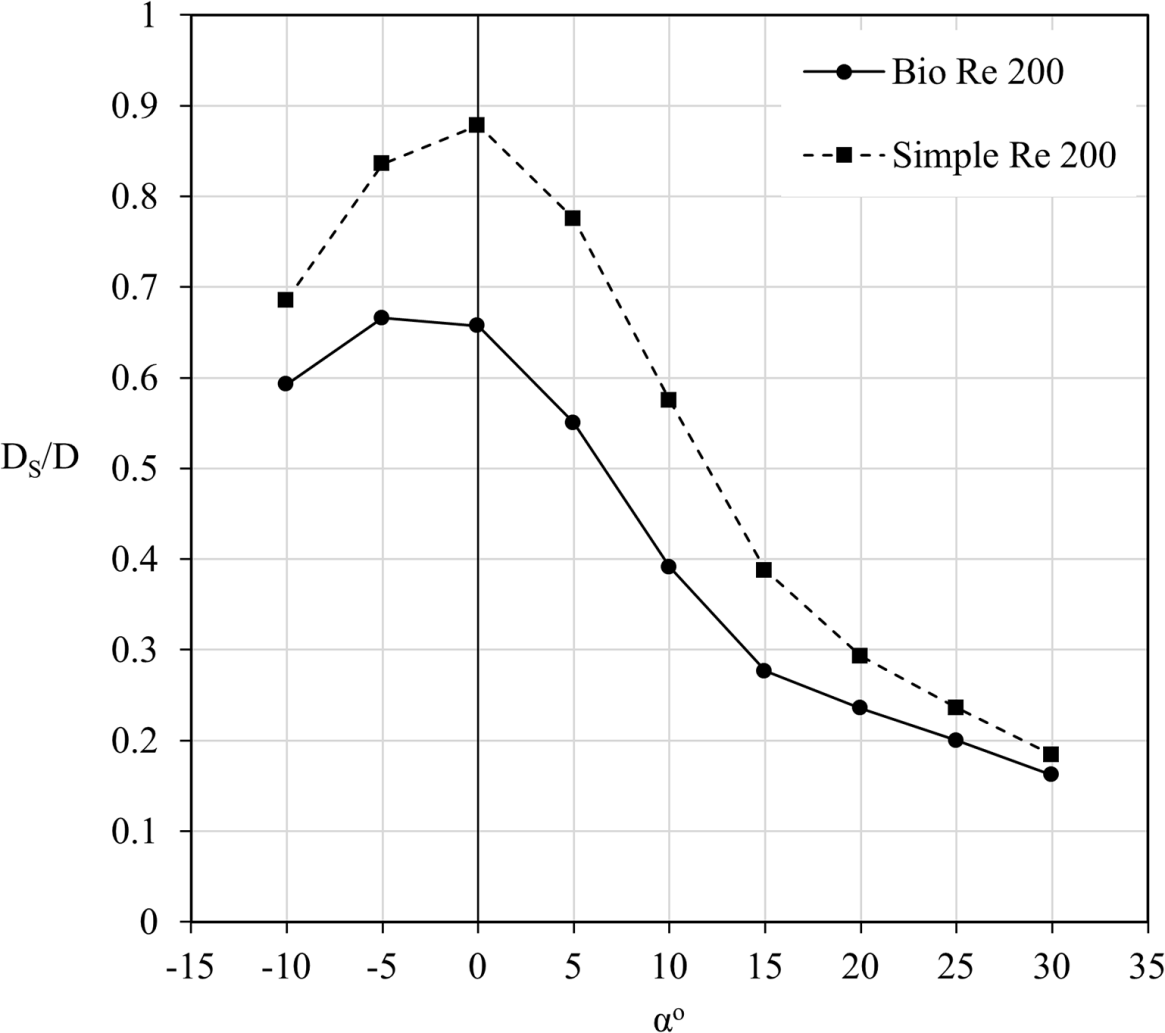
Proportional distribution of shear drag to total drag for the bio-realistic and simple wings. The proportion of shear drag to drag (D_S_/D) is plotted against angle of attack (α°) for both the bio-realistic and simple wings for a Reynolds number of 200. The simple wing maintains a noticeably higher proportion of shear drag, at lower angles of attack, however both wings present a very similar proportion of shear drag at the higher angles. This suggests that the corrugations of the bio-realistic wing have less effect on the overall drag at such angles.

Fig 4 additionally highlights a performance advantage for the simple wing over the bio-realistic version. At any of the tested Reynolds numbers, the simple wing exhibits increased lift over the bio-realistic wing, becoming more noticeable at angles of attack greater than 15°. The drag is also lower for the simple wing across the range of angles tested, however the difference in drag between the wings is almost negligible at 25° and above. By examining Fig 5, it is clear that the proportion of shear drag to total drag approaches very similar levels with increasing angle of attack for both wings. This suggests that the corrugations on the bio-realistic wing have a decreasing significance on the overall drag of the wing as the flow becomes increasingly dominated by the complete separation around the suction surface (as shown in a later section).

**Fig 6:**
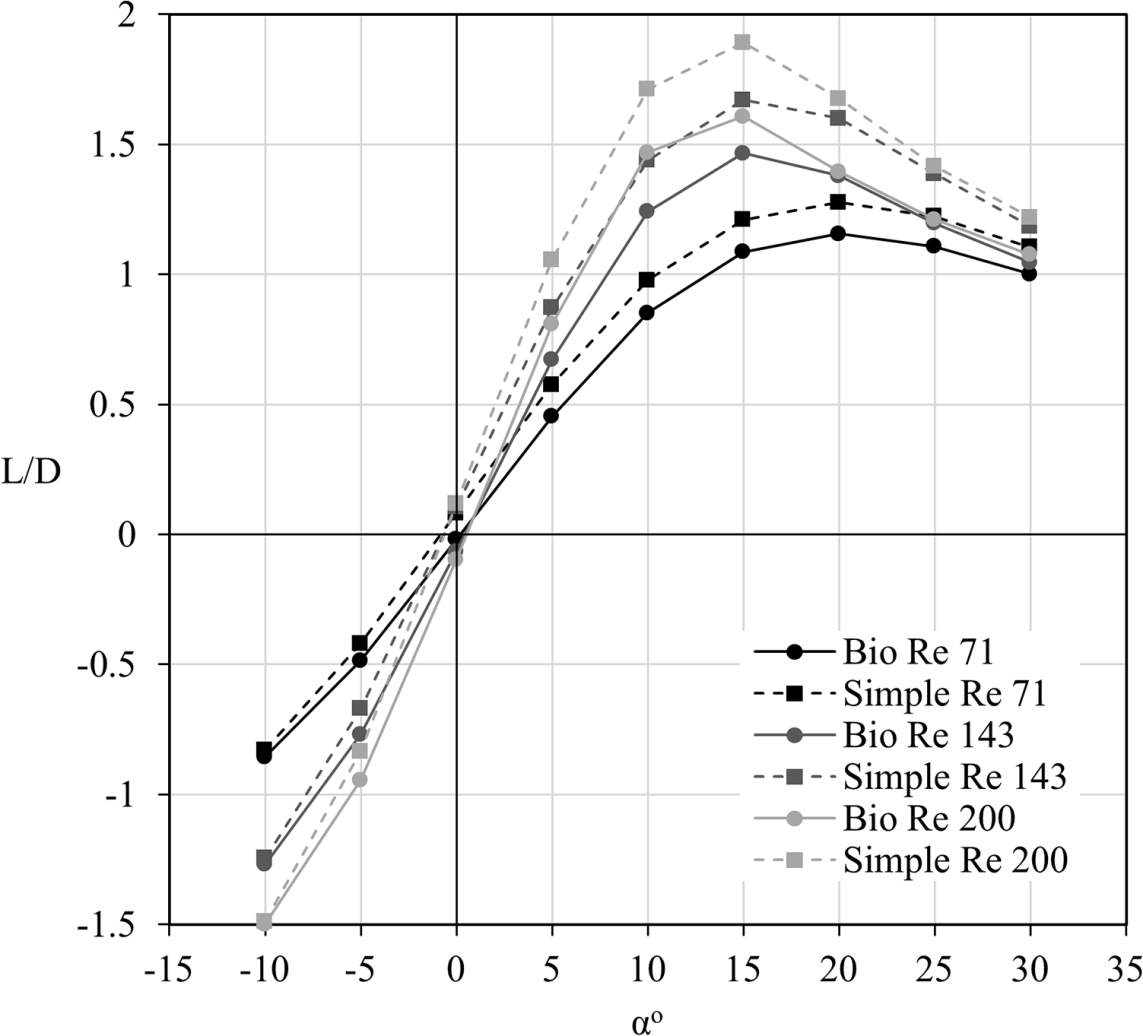
Lift to drag performance comparison between the bio-realistic and flat wings. The lift to drag (L/D) performance of both the bio-realistic (bio) and flat wings plotted against angle of attack (α°). The flat wing is shown to consistently have greater performance for all Reynolds numbers and angles of attack.

The flat wing had an improved gliding ratio (L/D) over the bio-realistic wing for all Reynolds numbers and angles of attack larger than -5° as shown in Fig 6. At -10°, both wings exhibited very similar performance. At best L/D, the flat wing outperformed the bio-realistic wing by 10.4%, 13.9% and 17.7% for Reynolds numbers 71, 143 and 200 respectively. As expected, increasing the Reynolds number improved the performance of both wings, due to the relative decreasing influence of viscous effects. A decline in overall efficiency was observed beyond 15° at Reynolds numbers of 143 and 200, whereas this decline is observed beyond 20° at a Reynolds number of 71.

#### Pressure distributions and three-dimensional effects

**Fig 7:**
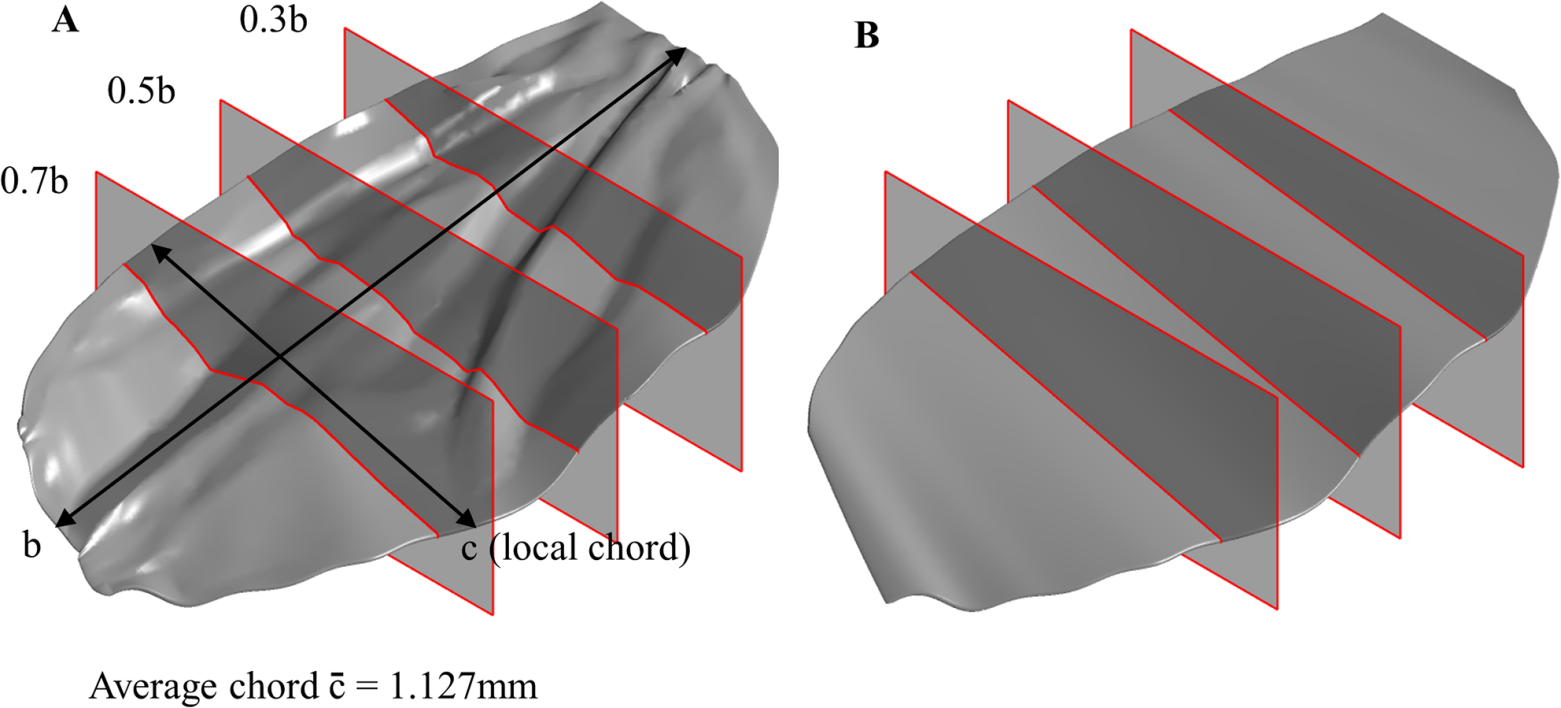
Cross-sections utilized for pressure plot comparisons. Cross sections taken at 0.3, 0.5, and 0.7 of the span, b, where c is the local chord length. These sections are utilised for comparison between A) the bio-realistic wing and B) the flat wing. Note that both wings share identical leading and trailing edges—the only difference is the corrugations.

If one simply relied upon the force coefficients to understand the effects of corrugations on wing performance, it would be an easy conclusion to state that the corrugated wing offers no performance advantage over the flat wing and indeed that a micro-air-vehicle being designed along similar principles would benefit from a flat wing as a result. However, the headline numbers for lift mask the more complex reality of the flow, as indicated by the pressure coefficients. The C_P_ plots are presented for a series of spanwise slices, taken at 0.3b, 0.5b and 0.7b as shown in Fig 7, and are accompanied by streaklines for simple and corrugated wings so that the shape of the wing at each section, and the nature of the wider flow-field, can be considered concurrently.

**Fig 8:**
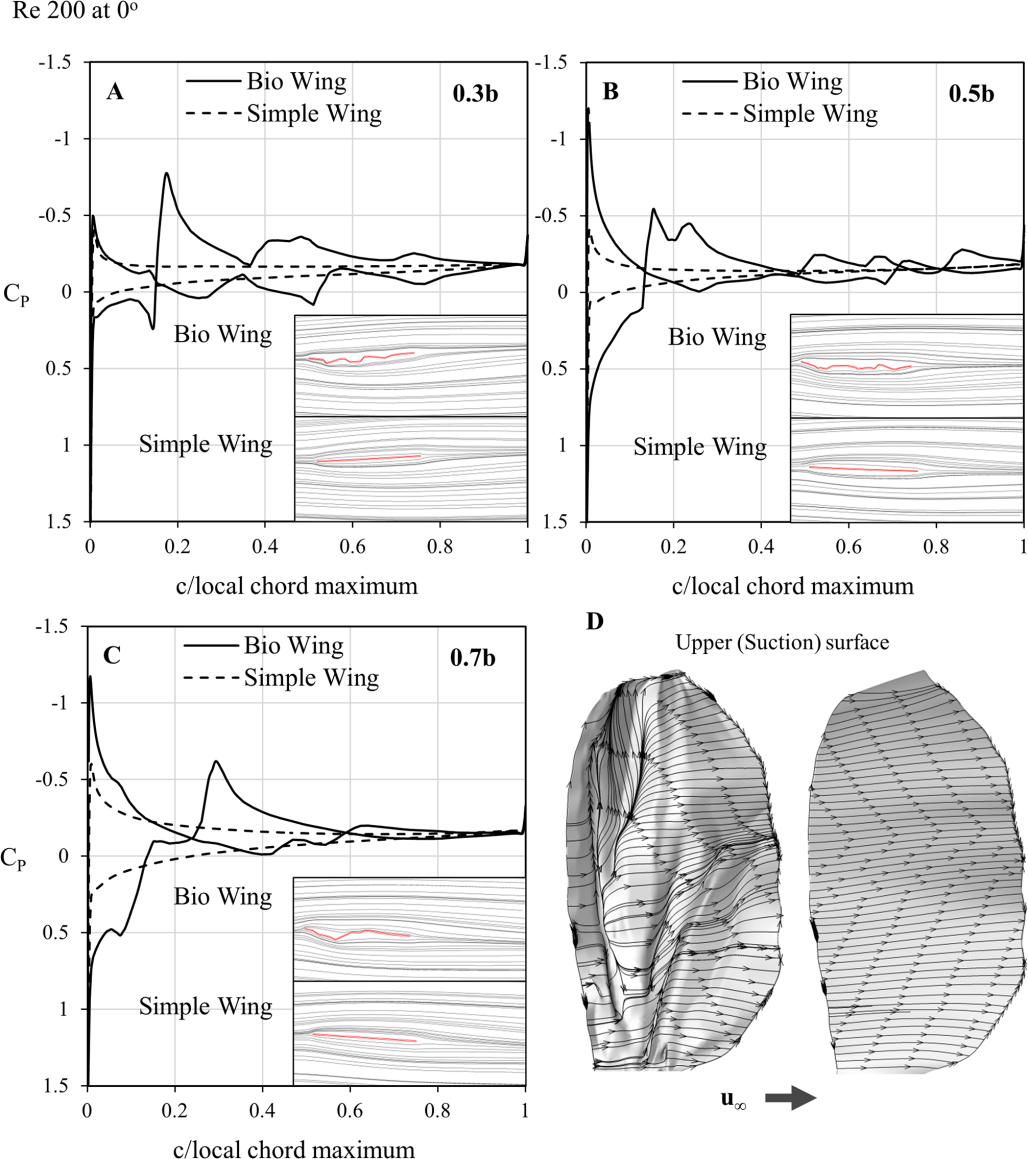
Coefficient of pressure and streakline comparisons at 0° of the bio-realistic and simple wings. Coefficient of pressure (C_P_) plot and streakline comparisons between the bio-realistic and simple wings at A) 0.3 span, B) 0.5 span and C) 0.7 span for a Reynolds number of 200 at an angle of attack of 0°. The fluctuations in pressure across the bio-realistic wing are due to the corrugations—while the coefficient of pressure rarely ever matches that of the flat wing, when integrated over the entire chord the forces correlate with those of the flat wing. D) presents a surface oil-flow of the velocity vectors, where U_∞_ is the freestream velocity direction. The surface oil-flow indicates that there exists some recirculation on the upper surface of the bio-realistic wing (left), which is not apparent on the flat wing (right). The maximum pressure coefficient has been truncated to 1.5 for clarity of the pressure distribution along the rest of the wing.

**Fig 9:**
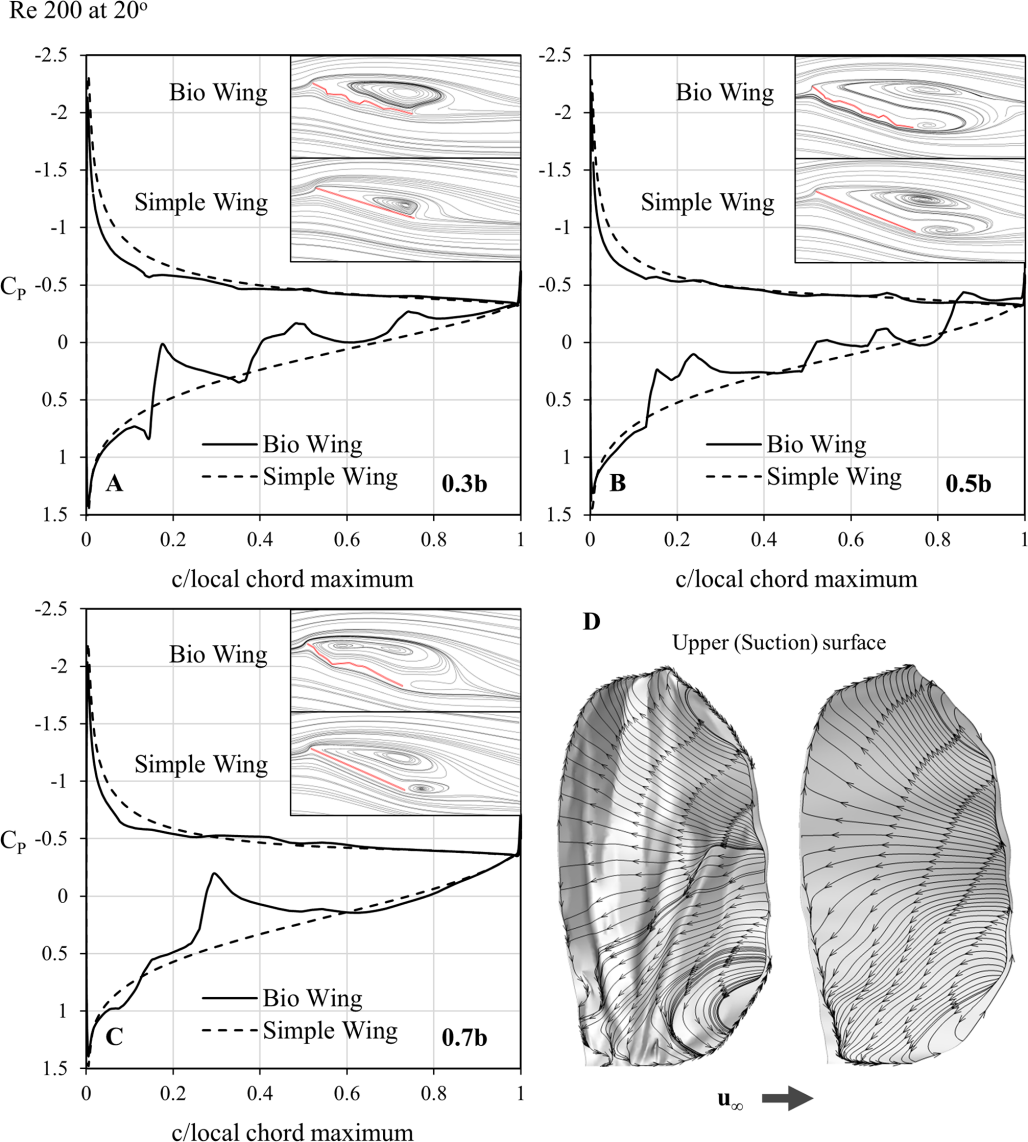
Coefficient of pressure and streakline comparisons at 20° of the bio-realistic and simple wings. Coefficient of pressure (C_P_) plot and streakline comparisons between the bio-realistic and simple wings at A) 0.3 span, B) 0.5 span and C) 0.7 span for a Reynolds number of 200 at an angle of attack of 20°. D) presents a surface oil-flow of the velocity vectors, where U_∞_ is the freestream velocity direction. Both wings now show signs of recirculation, indicated by the fully reversed flow of the surface oil-flow. Large vortices appear on both wings, however the bio-realistic wing consistently suffers a larger degree of separation. Due to this separation, the C_P_ plots of the upper surfaces match far more closely than the 0° case, and the major differences in force due to pressure appear to be driven primarily from the lower surface.

These images are shown in Fig 8 for 0° and Fig 9 for positive 20°. The Reynolds number of 200 was chosen as the effects are representative of those at lower Reynolds numbers as well.

Looking initially at the zero incidence case in Fig 8, it is notable that the pressure coefficient experienced by the leading edge stagnation in all cases is greater than 1. While unusual at high Reynolds numbers, pressure coefficients greater than 1 have been previously theoretically simulated on a sphere in very low Reynolds number cases [31, 32].

While the lift plots indicated a similar performance for the two types of wing, the pressure coefficients reveal that the corrugated wing sections experience considerable fluctuation in C_P_ due to the abrupt changes in geometry between veins. At any given individual point on the wing, the C_P_ is therefore unlikely to be anything like the simple counterpart, however integration across the surface yields a resultant force in close agreement with its simple counterpart.

At higher Reynolds numbers on other corrugated planforms, formation of significant near-stagnated regions in the natural valleys formed by the corrugations has been observed [21, 22] forming an approximate cambered shape if one follows the flow over the top of these recirculation cells. This increased thickness to the effective profile was expected to be responsible for the increase in drag noted for corrugated profiles compared to flat plates [13, 33]. However at Re = 200 and below, this feature is not as readily observable, with the flow able to better follow the contour of the wing with reasonably high fidelity when at 0° incidence in particular.

As shown in Fig 9, at 20°, separation occurs at the leading edge and the flow does not formally re-attach on the upper surface until the trailing edge—this is true of the simple wing as well as the bio-realistic wing, and the discrepancies in the magnitude and lateral extent of the larger-scale separated recirculation cells appears to be determined by the increased incident angle formed by the leading edge. The oncoming flow experiences a higher apparent angle of attack and therefore the flow around the forward part of the wing behaves as one would expect from the simple wing at a slightly higher angle. The re-attachment of the flow right at the trailing edge would indicate a strong requirement for increased stiffness in this region to cope with the repeated high-frequency load-cycling that would be experienced here in particular, though the vein structure sketched in Fig 1 indicates that this is provided almost entirely by the delineating feature rather than any spanwise support prior to the trailing edge.

At the inboard station of 0.3b, the corrugated wing most closely resembles the simple wing, and yet the recirculation above the upper surface is notably more extensive. Despite this, the sectional pressure coefficient indicates a less pronounced suction in this area with a lower-magnitude low-pressure suction surface. In examining the numerical oil-flow images that accompany the pressure distributions (Fig 9D) this relative discrepancy is explained by the more extensive spanwise component of the fully-reversed flow.

At the more outboard locations, the behavior of the flow next to the surface is remarkably similar between both types of wing. The C_P_ plots highlight that the loss of lift for the corrugated wing stems from the interruptions to higher pressure zones on the lower surface due to local pockets of lower pressure from flow turning. At the zero angle of attack, both upper and lower surfaces created local highs and lows from flow interactions, however the total separation on the upper surface negates this effect and the lower surface is dominant in determining overall performance of one wing relative to the other.

While recirculation pockets exist, as evident from the reversed flow seen in the surface oil-flow representation in Fig 8D, these do not significantly alter the effective profile of the wing until the more outboard region where the first corrugation is most pronounced. This is clear from the surface flow towards the tip which exhibits a clear fore and aft behavior difference at about the mid-chord, and in the pressure distribution at 0.7b this is reflected in the significant suction deficit which coincides with flow creating resultant high pressure on the upper surface at the same point at which flow on the underside is creating low pressure by navigating the corner.

**Fig 10:**
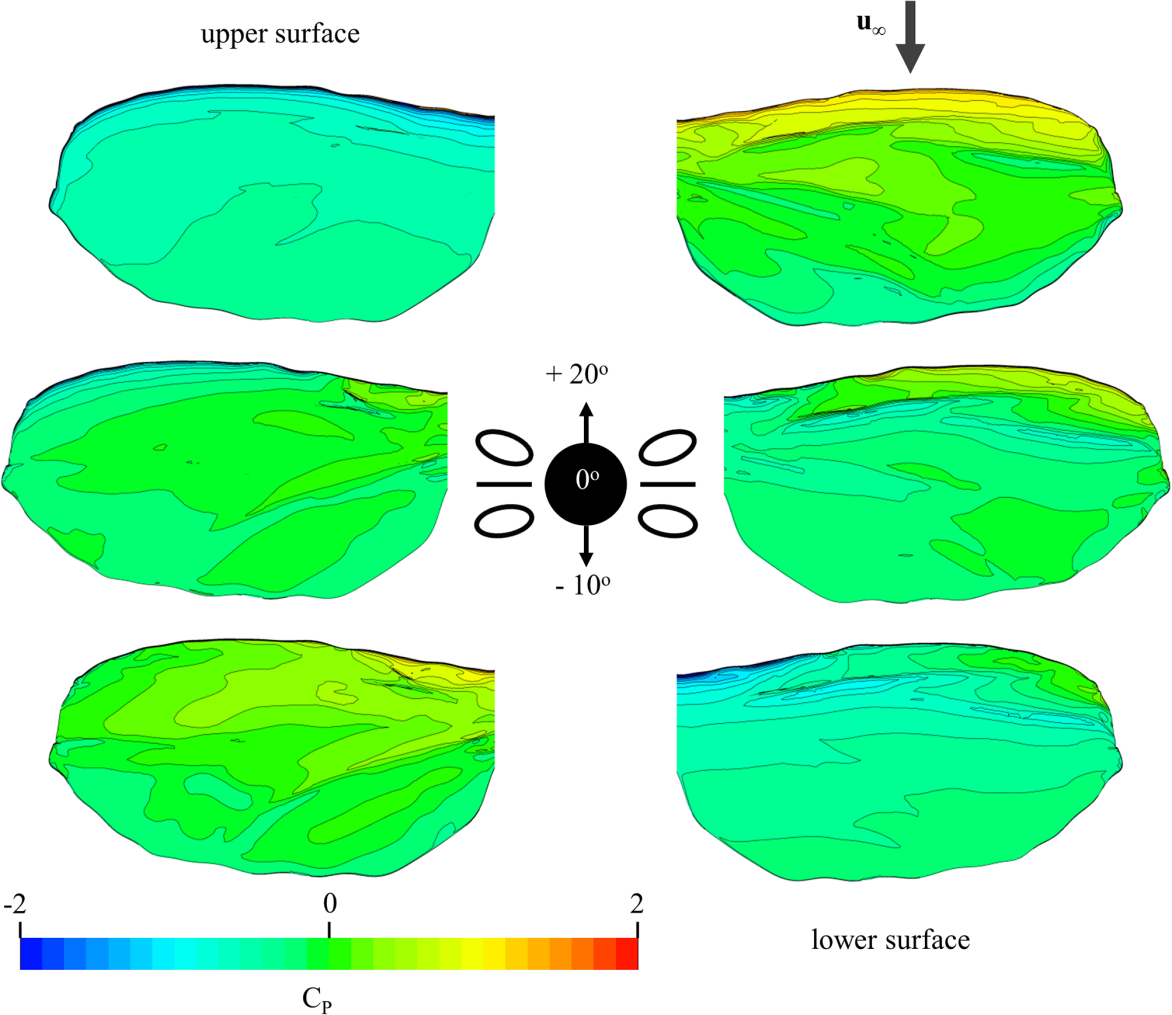
Pressure distribution along the bio-realistic wing at varying angles. Pressure distribution on the upper and lower surfaces of bio-realistic wing at angles of attack of 20°, 0° and -10°. The high three-dimensionality of the pressure distribution is significantly more evident when the surface of the wing is more directly in the path of the free stream.

The high three-dimensionality of the flow is exemplified by the pressure distribution of the corrugated wing shown in Fig 10. Observable are the local high and low pressure regions as induced by the corrugations, which were also noted in Fig 8 and Fig 9. One observable trend is that the pressure distribution is least homogenous when the corrugations are most directly in the path of the flow. For example, the upper surface pressure distribution for 20° presents a relatively smooth pressure transition from root to tip when compared to the lower angles of attack, which is expected due to the flow being mostly separated over the top of the wing (Fig 9). However, at this angle of attack, the lower surface is significantly within the flow path, and as such the pressure distribution is significantly three dimensional, being affected by the corrugations.

While Hord and Liang (2012) [33] investigated the suitability of a two-dimensional assumption when simulating flow around the corrugations of a dragonfly wing in gliding condition (non-flapping), by comparing to an extruded wing of aspect ratio 1, they concluded that the 8% difference in lift and drag was due to meshing variation rather than any flow parallel to the span of the wing (i.e. three dimensionality). This is obviously a feature of excessive simplification of an insect wing, as the present results indicate a very strong three-dimensionality of flow on the corrugated wing, partly due to the elliptical wing tip and associated effects also seen for the simple wing, and partly due to the irregular nature of the corrugations leading to a natural spanwise flow component at every point on the upper and lower surface. Given that a more comprehensive simulation of a flapping, aeroelastic wing would depend heavily on these features, consideration of the full realistic shape of the wing appears to be an important factor in determining the true flow behavior which could be produced by a corrugated wing.

#### Micro-CT

The fly’s thin wings are at the limit of spatial resolution achievable with polychromatic micro-tomography systems. Future micro-CT studies of insect wings should consider the use of contrast agents to enhance radio-opacity of the delicate wing structures [26, 34–38]. Due to the lack of such agents utilized in this study, information was missing from the initial scan as seen in Fig 1, and required interpolation from the available data when constructing a smooth, continuous surface for CFD use. This problem was somewhat compounded by the fact that it is more desirable to rotate the specimen a full 360° in the scanning process. Due to the limits of the post-processing software (CTVox) with regards to file size, it was decided that a 180° rotation would need to suffice.

Despite these difficulties, the wing still presents a far higher degree of accuracy when compared to previous studies [3–6, 20–22]. The complex corrugated structure is clearly visible, and matches the vein structure shown in Houle et al.’s (2003) [30] work, as seen in Fig 1.

## Conclusions

This paper covered a method by which a fruit-fly wing was converted from a micro-CT scan to a usable surface in CFD. By comparing this bio-realistic wing to a simplified flat wing, sources of potential numerical error were studied by examining both the high level performance of the wings and the detailed flow fields.

While both wings presented a three dimensionality in their flow field, the corrugations of the bio-realistic wing appeared to guide the formed vortices. From a purely aerodynamic point of view, the results suggest that the existence of corrugations is in fact a detriment to the performance of the wing operating at or below a Reynolds number of 200, agreeing with previous studies on gliding flight [21, 22].

Despite major differences in geometry, it was found that the simple wing provided near identical performance trends when purely examining lift and drag. However, the disparities in performance values, near wing flow-field shape and pressure distributions along the chord emphasize that the simple wing cannot provide the same detailed information. Thus, to complement the understanding of the mechanics behind insect flight, inclusion of the corrugations and complex 3D geometry is recommended.
